# Breakfast in Canada: Prevalence of Consumption, Contribution to Nutrient and Food Group Intakes, and Variability across Tertiles of Daily Diet Quality. A Study from the International Breakfast Research Initiative

**DOI:** 10.3390/nu10080985

**Published:** 2018-07-27

**Authors:** Susan I. Barr, Hassan Vatanparast, Jessica Smith

**Affiliations:** 1 Food, Nutrition and Health, University of British Columbia, Vancouver, BC V6T 1Z4, Canada; 2 College of Pharmacy and Nutrition and School of Public Health, University of Saskatchewan, Saskatoon, SK S7N 4Z2, Canada; vatanh@usask.ca; 3 Bell Institute of Health, Nutrition and Food Safety, General Mills, Minneapolis, MN 55427-3870, USA; Jessica.Smith@genmills.com

**Keywords:** diet surveys, nutrient intake, breakfast, diet quality, dietary assessment

## Abstract

This study used 24-h recall data from the nationally representative 2015 Canadian Community Health Survey-Nutrition to assess breakfast intake among Canadians aged 6–12 years (*n* = 2331), 13–17 years (*n* = 2026), 18–54 years (*n* = 7651), and 55+ years (*n* = 6279). Overall, 90% consumed breakfast; breakfast consumers reported higher intakes of energy and key nutrients and had higher daily diet quality scores assessed using the Nutrient-Rich Foods Index 9.3 (NRF 9.3). Among breakfast consumers (*n* = 16,484), breakfast contributed a mean of 389 kcal (1628 kJ) and 21.6% of daily energy intake. Relative to its contribution to energy, breakfast contributed higher intakes of fruit, whole grains, and fluid milk, as well as associated nutrients (e.g., carbohydrate, total sugars, fiber, calcium, and vitamin D). Among breakfast consumers classified by daily dietary quality (NRF 9.3 score), energy intake at breakfast did not differ across tertiles for either children or adults. However, intakes of key nutrients, fiber, and total sugars increased across tertiles, and among adults, intakes of saturated fat and sodium decreased. Mean intakes of fruit, whole grains, and fluid milk also increased across tertiles, as did the proportion of individuals consuming these foods; higher fruit and milk intakes may explain higher sugar intakes as diet quality increased. Promoting the consumption of these foods at breakfast could contribute to improved diet quality among Canadians.

## 1. Introduction

Over the last several decades, the contributions of breakfast to daily nutrient intakes and adequacy have been examined, as have the characteristics of those who do and do not consume breakfast [1,2,3,4,5,6,7,8,9]. Breakfast consumption has also been linked with a number of health and cognitive outcomes, although causal relationships have not yet been established [1,2,10,11,12,13,14,15,16,17]. 

We previously reported data on the differences in daily nutrient intake and the prevalence of nutrient inadequacy between Canadian breakfast consumers and non-consumers [4,5] using data from the nationally representative 2004 Canadian Community Health Survey (CCHS) [18]. At that time, we found that 10% of children and 11% of adults did not consume breakfast on the day of the 24-h recall. Breakfast consumers had higher intakes of energy and several key nutrients, as well as a lower prevalence of nutrient inadequacy. In 2015, the CCHS-Nutrition survey was repeated, using methods that were similar to those used in the 2004 CCHS [19]. This provided an opportunity to update the Canadian information and also to contribute data to the International Breakfast Research Initiative (IBRI), in which a harmonized approach using national dietary survey data from Canada, Denmark, France, Spain, the United Kingdom, and the United States is being used to study the nutritional impact of breakfast [2]. Accordingly, the objectives of the present study were as follows:

(1) To describe the context and prevalence of breakfast consumption in Canada;
(2) To compare daily energy and nutrient intakes of breakfast consumers and non-consumers;
(3) To describe the composition of breakfast and its contribution to daily nutrient intake and intake from key food groups; and
(4) Among breakfast consumers, to compare energy, nutrient, and food group intakes at breakfast by tertiles of daily dietary quality.

## 2. Materials and Methods

### 2.1. Data Source and Analytical Sample

The 2015 CCHS-Nutrition is a cross-sectional, nationally representative survey with a focus on nutrition [19,20]. Breakfast intake was self-reported and included all foods or beverages the respondents reported as having consumed at an eating occasion they identified as ‘breakfast’ during the survey’s 24-h recall. The target population for the survey included all individuals aged one year and above living in private dwellings in the 10 Canadian provinces, with a desired completed sample size of approximately (~)20,000 respondents representing ~98% of the Canadian population. Individuals who were full-time members of the Canadian Forces or who lived in the Territories, on reserves and other Aboriginal settlements, in some remote areas, or in institutions (e.g., prisons, care facilities) were not included in the target population. The overall response rate at the national level was 62% [19,20].

The multistage stratified cluster sampling strategy was designed to collect representative data, taking into account distributions based on age, sex, geography, and socioeconomic status. The data collection was completed in person by trained interviewers. The survey components included 24-h dietary recalls, a general health questionnaire to assess sociodemographic and lifestyle variables, and measured height and weight [19,20]. The 24-h recall was based on a modification of the automated multiple pass method [21] and was completed using a computer-assisted personal interview. This included a step in which the respondents reported the time of day they began eating or drinking each of the reported food items, as well as what they would call the eating occasion (e.g., breakfast, snack, dinner) for the food item. Children aged 6–11 years were interviewed for the general health questionnaire and 24-h recall in the presence of a parent or guardian, who could contribute to providing information. Respondents aged 12 years and above were interviewed on their own.

The present study included data from 18,287 respondents aged 6 years and above who were not pregnant or lactating and who were not extreme outliers for energy intake (extreme outliers were identified as those with intakes <200 or >8000 kcal/day; fewer than 0.03% of the sample were excluded on this basis). Although the 2015 CCHS-Nutrition ascertained use of nutritional supplements, our data reflected nutrient intakes from food alone. Dietary guidance both in Canada and globally encourages individuals to meet their nutritional requirements from food sources where possible, and therefore, we focused on intake from food.

The ethical approval for population surveys conducted by Statistics Canada, such as the 2015 CCHS-Nutrition, is based on the authority of the Statistics Act of Canada. The approval to conduct the analyses reported herein, and access to the CCHS data files, was provided by the Statistics Canada Research Data Centre Program [22]. Secondary analysis of these data was conducted in the present study.

### 2.2. Dietary Quality

To provide an estimate of diet quality, a modification of the Nutrient-Rich Foods Index 9.3 (NRF 9.3) was applied to the total daily diet. The NRF 9.3 was originally developed to compare the nutritional quality of individual foods per 100 kcal or standard serving [23]. It represented the sum of the percentage of daily values (DVs) of nine nutrients to encourage (protein, fiber, vitamin A, vitamin C, vitamin E, calcium, iron, magnesium, and potassium) minus the sum of the percentage of the maximum recommended values for three nutrients to limit (saturated fat, total or added sugar, and sodium). As proposed for use in the IBRI studies [2], the NRF 9.3 score reflects daily nutrient intakes (normalized to an energy intake of 2000 kcal (8.2 MJ), expressed as percentages of national or regional DVs. for food labeling purposes. In addition, given the emergence of vitamin D as a public health concern [24,25], all six IBRI countries replaced vitamin E with vitamin D in the list of nine nutrients to encourage. Percentage DVs. for these nine nutrients were truncated at 100 for intakes above the DV. For each of the three nutrients to limit, percentage DVs. were expressed as the percentage by which intake exceeded the DV (and accordingly, were 0 when intake was <DV). The maximum possible score was 900 points, reflecting a diet in which intakes per 2000 kcal were >DVs. for all nine nutrients to encourage and were <DVs. for all three nutrients to limit. The Canadian DVs. were used in the present study [26]. Because Canada has a DV for total sugars rather than added sugars and because data on added sugars are not available in the Canadian Nutrient File [27], total sugars were used as a nutrient to limit. Canada does not have a DV for protein, so we used a value of 10% of energy, which is the lower boundary of the acceptable macronutrient distribution range [28]. Thus, the DVs. used to calculate the NRF 9.3 were as follows: protein, 50 g; fiber, 28 g; vitamin A, 900 μg retinol activity equivalents (RAE); vitamin C, 90 mg; vitamin D, 20 μg; calcium, 1300 mg; iron, 18 mg; magnesium, 420 mg; potassium, 4700 mg; saturated/trans fat, 20 g; total sugars, 100 g; and sodium, 2300 mg.

### 2.3. Food Group Intakes

Foods and beverages in the Canadian Nutrient File are classified into food groups and subgroups using the framework provided by Canada’s Food Guide [29,30], and these groupings were used to report the types of foods consumed. The four major food groups are vegetables and fruits, grain products, milk and alternatives, and meat and alternatives; an “other foods” group consists of items that fall outside the four core food groups, such as fats and oils, alcohol, and items high in fat or sugar, such as sweets, sodas, and salty snack foods. Subgroups of the major food groups that reflect specific directional advice provided in the Food Guide (e.g., “make at least half of your grain products whole grain each day”) are also identified. For this analysis, subgroups included whole fruit, fruit juice, whole grains, non-whole grains, fluid milk, and other milk products. Some items, primarily those identified as “recipes” (e.g., an omelet made with eggs, ham, vegetables, and cheese), are not classified [30].

### 2.4. Statistical Analysis 

All statistical analyses were performed using SAS (Version 9.4, SAS Institute) software. In all analyses, we followed Statistics Canada recommendations on weighting and bootstrapping to obtain estimates at a population level [31]. Values are reported as percentages ± standard errors (SE) for categorical variables and means ± SEs for continuous variables. Alpha was set at 0.05 in all analyses. For post hoc tests, multiple comparisons with the best (MCB) and a Hsu-adjusted α = 0.025 were applied [32].

#### 2.4.1. Prevalence and Context of Breakfast Consumption

Descriptive analyses included the prevalence of breakfast consumption by age group (children (6–12 years), teens (13–17 years), adults (18–54 years), and older adults (55+ years)), sex, location, weekday/weekend day, and time of day.

#### 2.4.2. Daily Energy and Nutrient Intakes of Breakfast Consumers and Non-Consumers

Sociodemographic and lifestyle characteristics of breakfast consumers and non-consumers were compared using chi-square tests for categorical variables and F-statistics for continuous variables (age, body mass index (BMI; kg/m^2^), BMI *z*-score, daily energy intake). The categorical variables were dichotomized from questionnaire data as follows: sex (male, female); ethnicity (Caucasian, non-Caucasian); supplement use (any use of a nutritional supplement in the past 30 days versus no use); smoking status (current smoker versus past/never smoker); education (university graduate versus lower level of educational attainment); marital status (married/cohabiting versus single, divorced, or widowed); food security (food secure versus food insecure); weight status (overweight/obese versus normal weight); area of residence (urban versus rural); and immigration status (immigrant to Canada versus born in Canada). Variables that differed significantly between breakfast consumers and non-consumers were included as covariates in regression models comparing daily energy intakes, nutrient intakes, food group intakes, and diet quality using analysis of covariance (ANCOVA). 

#### 2.4.3. Breakfast Composition and Contribution of Breakfast to Daily Nutrient and Food Group Intake

These analyses were limited to breakfast consumers. The descriptive analyses included the following: mean nutrient intake at breakfast by age group and for all breakfast consumers aged 6 years and above; the mean number of servings of foods from the key food groups and food subgroups of Canada’s Food Guide [29,30], as well as the percentage of individuals consuming these foods at breakfast; and the percentage contribution of breakfast to daily intakes of nutrients and food groups. ANOVA was used to test for differences across age groups.

#### 2.4.4. Comparison of Energy, Nutrient, and Food Group Intakes at Breakfast by Tertiles of Daily Dietary Quality

Breakfast consumers (children/teens aged 6–17 years and adults ≥ 18 years) were classified into tertiles of daily diet quality based on their NRF 9.3 score. Sociodemographic and lifestyle characteristics were compared across tertiles using chi-square tests and F-statistics, and variables that differed significantly were included as covariates in subsequent regression models comparing nutrient and food group intakes at breakfast across tertiles of diet quality.

## 3. Results

### 3.1. Prevalence and Context of Breakfast Consumption

Overall, on any given day, 90.0% ± 0.5% of Canadians reported consuming breakfast, and consumption did not differ between weekdays and weekend days (89.8% ± 0.6% vs. 90.2% ± 0.7%, *p* = 0.70). Breakfast was consumed before 7:00 a.m. by 13.5% ± 0.5%, between 7:00–8:59 a.m. by 50.0% ± 0.8%, between 9:00–10:59 a.m. by 31.1% ± 0.7%, and at 11:00 a.m. or later by 5.4% ± 0.3%. Women were more likely to eat breakfast than men (91.4% ± 0.6% vs. 88.6% ± 0.7%, *p* = 0.002). The prevalence of breakfast consumption was highest in children aged 6–12 years (96.9% ± 0.4%), followed by older adults aged 55+ years (92.5% ± 0.7%), and then by adolescents aged 13–17 years (87.9% ± 1.1%) and younger adults aged 18–54 years (87.6% ± 0.8%). Among breakfast consumers, 85.7% ± 0.5% of breakfasts were consumed at home, with adults aged 18–54 years the least likely to consume breakfast at home (81.1% ± 0.9%) and children the most likely to do so (90.9% ± 0.9%). Other sites for eating breakfast included at work (4.7% ± 0.3%), at a restaurant including fast-food restaurants (4.6% ± 0.3%), and other locations (e.g., in the car, at someone else’s home; 5.0% ± 0.3%). 

Sociodemographic and lifestyle characteristics of children/teen and adult breakfast consumers and non-consumers are compared in Table 1. Among children/teens, breakfast consumers were significantly younger, less likely to smoke, more likely to have a household member with a university degree, and less likely to be overweight or obese (although BMI *z*-scores did not differ). Among adults, breakfast consumers were significantly older, less likely to be male or to smoke, and more likely to use supplements, be a university graduate, be married or cohabiting, be food secure, and be an immigrant to Canada. Accordingly, these variables were included in regression models comparing the daily intakes of breakfast consumers and non-consumers.

### 3.2. Daily Intakes of Breakfast Consumers and Non-Consumers 

Table 2 displays unadjusted mean energy and nutrient intakes of breakfast consumers and non-consumers, for the entire Canadian population aged 6 years and above and by age group. When analyzed using regression models that included energy and sociodemographic covariates, breakfast consumers as a group had higher absolute carbohydrate intakes, substantially higher intakes of fiber, similar protein intakes, and a modest difference in total fat intake, compared with non-consumers. The percentage energy from carbohydrate was higher in breakfast consumers, while the percentage energy from protein and total fat did not differ. Breakfast consumers as a group also had significantly higher intakes of the following vitamins and minerals: thiamin, riboflavin, folate and folic acid, vitamin C, iron, magnesium, and potassium. Furthermore, the NRF 9.3 score was 19% higher in breakfast consumers. When analyzed by age group, breakfast consumers had higher intakes than non-consumers in all four age groups of fiber, vitamin C, and magnesium and, in three of four age groups, higher intakes of thiamin and iron and a higher NRF 9.3 score than non-consumers. Teens aged 13–17 years had the greatest number of differences between consumers and non-consumers: in addition to a higher NRF 9.3 score, breakfast consumers had higher intakes of fiber, vitamin A, thiamin, riboflavin, vitamin B6, vitamin B12, vitamin C, vitamin D, calcium, iron, magnesium, potassium, and zinc, as well as lower intakes of cholesterol.

### 3.3. Composition of Breakfast and Contribution of Breakfast to Daily Nutrient and Food Group Intakes

#### 3.3.1. Energy and Nutrient Intakes at Breakfast

Table 3 presents data on the energy and nutrient composition of breakfast for all Canadian breakfast consumers age 6 years and above and by age group. For all ages, breakfast provided about 400 kcal (~1600 kJ), with intakes lowest in children and highest in teens. With the exception of vitamin B6 and vitamin C, breakfast nutrient intakes differed significantly across age groups. In most cases, age-related differences in nutrient intakes were associated with breakfast energy intake and so were lowest in children or older adults (the two groups with lower breakfast energy intakes) and highest in teens or adults aged 18–54, who had higher breakfast energy intakes. However, there were a few exceptions: children had the highest intakes of carbohydrate and total sugars expressed as a percentage of breakfast energy, adults aged 18–54 years had the lowest intakes of iron, and older adults had the highest intakes of fiber, magnesium, and potassium.

#### 3.3.2. Contribution of Breakfast to Daily Nutrient Intakes

**Figure 1:**
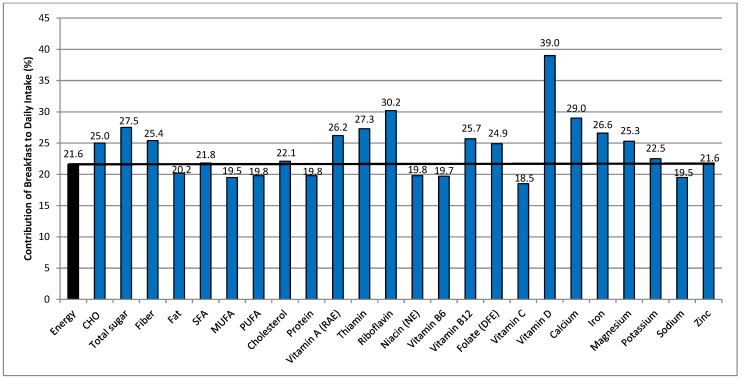
Contribution of nutrient intakes at breakfast to total daily nutrient intakes of Canadian breakfast consumers aged 6 years and above (*n* = 16,484). The horizontal line is the percent of daily energy intake consumed at breakfast. CHO: carbohydrate; DFE: dietary folate equivalents; MUFA: monounsaturated fatty acids; NE: niacin equivalents; PUFA: polyunsaturated fatty acids; and RAE: retinol activity equivalents.

The contributions of breakfast to daily nutrient intakes of Canadians aged 6 years and above are shown graphically in Figure 1. Breakfast contributed an average 21.6% to daily energy intake and relative to energy, made higher contributions (i.e., >1% greater than its contribution to energy) to daily carbohydrate, total sugars, fiber, vitamin A, thiamin, riboflavin, vitamin B12, folate, Vitamin D, calcium, iron, and magnesium intakes. It provided lower contributions (>1% less than the energy contribution) to total fat, monounsaturated fatty acids (MUFA), polyunsaturated fatty acids (PUFA), protein, niacin, vitamin B6, vitamin C, and sodium intakes. Proportional contributions (within ±1% of the energy contribution) were provided for saturated fat, cholesterol, potassium, and zinc intakes. When examined by age group (see Figures S1 and S2), the contribution of breakfast to daily energy intake increased across age groups, from 19.7% in children 6–12 years to 22.7% in older adults. However, generally similar patterns to those reported for the entire population were observed in terms of nutrients provided in higher, lower, or proportional amounts relative to energy.

#### 3.3.3. Contribution of Breakfast to Daily Food Group Intakes

Table S1 shows the proportion of children/teens and adults consuming food items from the food groups, key subgroups, and other foods as well as the mean percentages of breakfast energy and nutrients provided by those food groups and subgroups. Taken together, these categories contributed ~80% of breakfast energy intake in both children and adults, as well as 62%–84% of breakfast intakes of nutrients included in the NRF 9.3. Foods that were not classified (primarily ‘recipe’ items) [30] would have contributed the remaining ~20% to breakfast energy intake.

**Figure 2:**
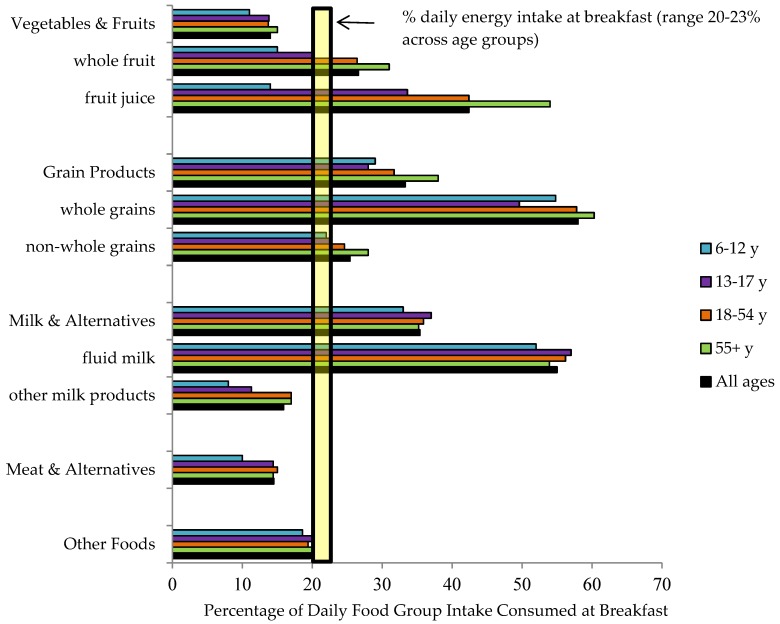
Contribution of breakfast to daily intakes of food groups and subgroups of Canadian breakfast consumers aged 6 years and above (*n* = 16,484) by age group and for all ages combined.

The contributions of breakfast to daily intakes of the four major food groups, key subgroups, and other foods are shown in Figure 2 by age group and for all ages combined. Relative to its contribution to daily energy, which ranged from ~20–23% across age groups, breakfast provided lower contributions to daily intakes from the vegetable and fruit and the meat and alternative groups, higher contributions to daily intakes of grain products and milk and alternatives, and proportional contributions to daily intakes of other foods. This pattern was observed in all age groups. When food subgroups were examined, breakfast made generally proportional contributions to daily intakes of non-whole grains (an average of 25%) but contributed an average of 58% of whole-grain intakes. Similarly, on average, only 16% of daily intakes of other milk products (primarily cheese and yogurt) were consumed at breakfast, compared with an average of 54% of fluid milk intake. These observations were also consistent across age groups. In contrast, clear differences across age groups were evident for intakes of whole fruit and fruit juice, with breakfast contributing 15% and 14% of children’s daily whole fruit and fruit juice intakes versus 31% and 54% of older adults’ daily whole fruit and fruit juice intakes.

#### 3.3.4. Contribution of Breakfast to Daily Food Group Intakes

Table S1 shows the proportion of children/teens and adults consuming food items from the food groups, key subgroups, and other foods as well as the mean percentages of breakfast energy and nutrients provided by those food groups and subgroups. Taken together, these categories contributed ~80% of breakfast energy intake in both children and adults, as well as 62%–84% of breakfast intakes of nutrients included in the NRF 9.3. Foods that were not classified (primarily ‘recipe’ items) [30] would have contributed the remaining ~20% to breakfast energy intake.

The contributions of breakfast to daily intakes of the four major food groups, key subgroups, and other foods are shown in Figure 2 by age group and for all ages combined. Relative to its contribution to daily energy, which ranged from ~20–23% across age groups, breakfast provided lower contributions to daily intakes from the vegetable and fruit and the meat and alternative groups, higher contributions to daily intakes of grain products and milk and alternatives, and proportional contributions to daily intakes of other foods. This pattern was observed in all age groups. When food subgroups were examined, breakfast made generally proportional contributions to daily intakes of non-whole grains (an average of 25%) but contributed an average of 58% of whole-grain intakes. Similarly, on average, only 16% of daily intakes of other milk products (primarily cheese and yogurt) were consumed at breakfast, compared with an average of 54% of fluid milk intake. These observations were also consistent across age groups. In contrast, clear differences across age groups were evident for intakes of whole fruit and fruit juice, with breakfast contributing 15% and 14% of children’s daily whole fruit and fruit juice intakes versus 31% and 54% of older adults’ daily whole fruit and fruit juice intakes.

### 3.4. Energy, Nutrient, and Food Group Intakes at Breakfast by Tertiles of Daily Dietary Quality

The breakfast consumers were divided into tertiles of daily dietary quality based on the NRF 9.3 score, and sociodemographic and lifestyle variables were compared across tertiles for children and teens aged 6–17 years and for adults aged 18 years and above (see Table S2). Among children and teens, those with higher diet quality were younger, less likely to smoke, more likely to have a household member who had graduated from university, more likely to be food secure, and more likely to reside in an urban area. Among adults, those with higher diet quality were older and were more likely to be women, to use dietary supplements, to have graduated from university, to be food secure, to have a lower BMI, and to be an immigrant to Canada. They were also less likely to smoke. Accordingly, these variables were included as covariates in regression models examining differences in intake across NRF 9.3 tertiles. In both children and adults, those with higher diet quality reported lower daily energy intakes.

Table 4 shows that energy intake at breakfast did not differ across daily NRF 9.3 tertiles. However, the proportion of daily energy provided at breakfast increased across tertiles of diet quality in adults; in children, it was higher in both upper tertiles compared with the lowest tertile. In adults, breakfast intakes of all nutrients except folate varied across tertiles, in most cases in the expected direction: intakes of carbohydrate, fiber, protein, vitamin A, thiamin, riboflavin, niacin, vitamin B6, vitamin B12, vitamins C and D, calcium, iron, magnesium, potassium, and zinc were highest in those with the highest daily diet quality (tertile 3), while intakes of fat, saturated fat, monounsaturates, polyunsaturates, cholesterol, and sodium were lowest in tertile 3. The one unexpected observation was that intakes of total sugars were highest in tertile 3. In children, the highest breakfast intakes were observed in tertile 3 for fiber, vitamin A, thiamin, riboflavin, vitamin B6, vitamin B12, vitamin D, calcium, iron, magnesium, potassium, and zinc. The total sugar intake was highest in tertile 2, although intakes in tertiles 2 and 3 were similar. Intakes at breakfast of protein, fat, saturated, monounsaturates, polyunsaturates, cholesterol, niacin, folate, vitamin C, and sodium did not differ significantly across tertiles, although similar directional trends to those seen in adults were observed. 

The mean intakes at breakfast from food groups and subgroups (averaged over all members of a tertile) and the proportions of children/teens and adults per tertile consuming foods from each of the food groups are shown in Table 5. Among both children and adults, the mean number of breakfast servings from the grain products and meat and alternatives groups did not differ across the daily NRF 9.3 tertiles. Those classified in tertile 1 (reflecting the lowest daily diet quality) had the lowest number of servings at breakfast from the vegetables and fruit group (and the whole fruit and fruit juice subgroups), the milk and alternatives group (and the fluid milk subgroup), and the whole grains subgroup. They also had the highest number of servings from the non-whole grains subgroup and the greatest energy intake from other foods. In contrast, those classified in tertile 3 showed the opposite patterns.

The data on the proportion of consumers of each food group across all three tertiles indicated that grain products (primarily non-whole grains) were consumed at breakfast by 80–90% of children and adults, milk and alternatives (primarily fluid milk) by 58–78% of children and 50–68% of adults, vegetables and fruit by 28–39% of children and 30–56% of adults, and meat and alternatives by about 25% of children and 37% of adults. Other foods were included in 50–68% of children’s breakfasts and 65–74% of adults’ breakfasts. Trends across tertiles in the proportions of individuals consuming items from food groups and subgroups were generally parallel to those observed for mean intakes. In both children and adults, tertile 3 had the highest percentages of individuals consuming vegetables and fruit, whole fruit, whole grains, milk and alternatives, and fluid milk at breakfast and the lowest percentages who consumed non-whole grains, other milk products, and other foods at breakfast.

Taken together, the data suggest that the proportion consuming a food group contributed much of the variability to the changes in mean serving size across tertiles, and that there was relatively modest variation in the serving size per consumer of the food group. For example, among children, the mean intake of whole grains at breakfast increased from 0.23 to 0.45 to 0.66 servings across the NRF 9.3 tertiles, while the proportion of consumers increased from 16.6% to 29.3% to 43.2%. Thus, the mean intake per whole grain consumer was relatively similar at 1.4 (0.23/0.166), 1.4 (0.45/0.293), and 1.5 (0.66/0.432) servings for tertiles 1–3, respectively. Similar calculations for adults revealed mean intakes per whole grain consumer of 1.65, 1.61, and 1.54 servings for tertiles 1–3. The other foods category, however, was an exception: energy intake per consumer declined noticeably across tertiles in both children (91, 79, and 51 kcal in tertiles 1–3, respectively) and adults (100, 81, and 60 kcal in tertiles 1–3, respectively).

## 4. Discussion

### 4.1. Prevalence and Context of Breakfast Consumption

In this large, nationally representative sample of Canadian children and adults, we found that 90% reported consuming breakfast on any given day, a proportion which has not changed since 2004 [4,5]. The prevalence of skipping breakfast in Canada (10%) appears substantially lower than in the United States, where it is close to 20% among both children and adults [6,7]; why this should be the case is not readily apparent. Canadian breakfast consumers and non-consumers differed with regard to a number of sociodemographic variables, which has been noted elsewhere [4,5,8,9]. As a group, breakfast non-consumers differed in age (they were older among children and adolescents and younger among adults), had characteristics suggestive of lower socioeconomic status (e.g., lower educational attainment and a lower prevalence of food security), and appeared to be less health-conscious (e.g., were more likely to smoke and less likely to use nutrition supplements). We did not observe ethnic differences between consumers and non-consumers but noted that the proportion of adult immigrants to Canada was higher among breakfast consumers compared with non-consumers, suggestive of a “healthy immigrant” effect [33].

Although eating breakfast away from home is described as a growing trend in North America [34], the vast majority of Canadian breakfasts were consumed at home, with fewer than 5% reporting that they ate breakfast at a restaurant. This suggests that the nutritional quality of the breakfast meal is largely determined by the individual consumer (and/or by other members of their household), making it a relevant target for intervention.

### 4.2. Daily Intakes of Breakfast Consumers and Non-Consumers

Significant differences in nutrient intake and adequacy between breakfast consumers and non-consumers have been reported in many previous studies (e.g., [2,4,5,6,7]), and our present data confirm these observations. We also observed a substantial difference in the daily NRF 9.3 score, which averaged 19.4% higher among breakfast consumers. It should be noted that this difference was not due to the lower reported energy intakes of non-consumers (which would lead to lower nutrient intakes if diet quality did not differ), as the daily NRF 9.3 score is normalized to an intake of 2000 kcal. When examining the data by age group, we found that teens aged 13–17 had the greatest number of differences in daily nutrient intakes between breakfast consumers and non-consumers. Nutrients that differed significantly included vitamin A, vitamin D, calcium, magnesium, potassium, and fiber, all of which have been identified as nutrients of concern for Canadian adolescents [35]. This age range is critical for building peak bone mass, to which calcium and vitamin D contribute [36]. As has been reported in other studies [5,9,37,38], there was also a substantial decrease in the prevalence of breakfast consumption between childhood and the teen years, suggesting that efforts directed towards encouraging breakfast consumption in this age group could be beneficial. 

### 4.3. Composition of Breakfast and Contribution of Breakfast to Daily Nutrient and Food Group Intakes

Canadian breakfasts provided an average of almost 400 kcal (~1600 kJ) kcal and ~22% of daily energy intake. This is somewhat higher than the average of ~17% of daily energy reported for the United States [39] but is within the range of 15–25% of daily energy that some have suggested as an appropriate intake at breakfast [3]. For all age groups, breakfast overcontributed (relative to energy) to intakes of several key nutrients of concern in the Canadian diet, including fiber, vitamin A, vitamin D, calcium, and magnesium. Particularly notable was the finding that it provided 39% of daily vitamin D intake, although it should be mentioned that the average daily intake of ~5 mcg was well below current recommended intakes of 15–20 mcg [40]. The data on serum 25-OH vitamin D values indicate that seasonal sunlight exposure likely makes substantive contributions to the vitamin D status of most Canadians [41], as the proportion with inadequacy based on serum 25-OH vitamin D concentrations is considerably lower than what would be expected based on dietary intakes. Despite this, low vitamin D status remains a concern for a proportion of the population and accordingly, Health Canada is proposing to approximately double current mandatory fortification levels in milk and margarine to 2 μg/100 mL and 26 μg/100 mL, respectively, effective in 2022 [42].

When breakfast intakes were expressed relative to breakfast’s contribution to daily energy intake, breakfasts consumed by all age groups contributed positively to intakes of milk and alternatives and grain products (especially whole grains) but under-contributed to intakes from the vegetables and fruits and meat and alternatives food groups. Canadian diets generally provide intakes of meat and alternatives and grain products that are close to the recommendations of Canada’s Food Guide [43] (although intakes of whole grains are considerably less than the recommended 50% of grain products [44]) while intakes from the vegetables and fruit and the milk and alternatives food groups generally fall below recommendations [43]. Thus, intakes of food groups at breakfast appeared to help mitigate observed shortfalls in meeting food group recommendations, although this was not true for the total vegetables and fruit group: while breakfast contributed positively to intakes of fruit and fruit juice relative to the rest of the day for the population as a whole, intakes of vegetables at breakfast were extremely low (data not shown; mean intake at breakfast was 3% of total daily vegetable intake). Young children, however, consumed lower proportions of their daily fruit juice and whole fruit intakes at breakfast than other age groups, suggesting that they are more likely than older age groups to consume these items at other eating occasions during the day (e.g., snacks). 

### 4.4. Energy, Nutrient, and Food Group Intakes at Breakfast by Tertiles of Daily Dietary Quality 

Like all countries participating in the IBRI [2], we used the NRF 9.3 score to stratify breakfast consumers by daily dietary quality and to assess the composition of breakfasts consumed by those with higher, moderate or lower daily diet quality. Unlike some other indices of diet quality (e.g., Healthy Eating Index, Mediterranean Diet Score), the NRF 9.3 is based on the intake of nutrients rather than food groups, which makes it more appropriate in the context of the IBRI, as classifications of food groups vary considerably across the six participating countries [2]. 

When children and adults were classified into tertiles of daily dietary quality, we observed a number of sociodemographic differences across tertiles that were similar to those detected between breakfast consumers and non-consumers. For example, among adults, breakfast non-consumers (versus consumers) and those in the lowest tertile (versus higher tertiles) of daily dietary quality were younger, more likely to be male and to smoke, and less likely to use nutritional supplements, to have graduated from university, to be food secure, and to be an immigrant to Canada. These observations emphasize the strong associations observed between sociodemographics, health behaviors, and health outcomes [45]. 

Although breakfast energy intakes did not differ across tertiles of daily diet quality, we observed the expected gradations in nutrient intakes: intakes of the NRF 9.3 nutrients to encourage increased, as did intakes of other vitamins and minerals not included in the NRF 9.3 score (e.g., thiamin, riboflavin, niacin, vitamin B6, vitamin B12, and zinc). Moreover, intakes of two of the three NRF 9.3 nutrients to limit (saturated fat and sodium) showed the expected decrease across tertiles. In terms of foods, intakes of fruit, whole grains, and fluid milk increased across tertiles, while intakes of other foods decreased. However, for both children and adults, those in the upper tertiles had total sugar intakes at breakfast that were 3–4 g higher than those in the lowest tertile, which contrasts with the expected lower sugar intake in higher quality diets. This may be due, at least in part, to the fact that we used total sugars as a nutrient to limit rather than added sugars when determining the NRF 9.3 score, as data on added sugars are not available in the Canadian Nutrient File. The increase of 3–4 g in total sugar intake across tertiles can be compared to what would be expected based on differences in mean intakes of fluid milk, whole fruit, and fruit juice across tertiles (data shown in Table 5). Among adults, mean intakes of fruit (whole fruit and fruit juice combined) and fluid milk in the third tertile were 0.82 and 0.38 servings, respectively, compared with 0.34 and 0.18 servings, respectively, in the first tertile. Thus, mean breakfast intakes of fruit and fluid milk were 0.48 and 0.2 servings higher in the third tertile, respectively. Based on estimated total sugar content of ~10–15 g per serving of fruit and 12 g per serving of fluid milk, the expected difference in sugar intake would be about 8.5 g, which is considerably greater than the observed difference of 4 g. Similar data for children (differences of 0.25 servings of fruit and 0.35 servings of milk between the first and third tertiles) would predict a difference in sugar intake of about 7 g. The higher predicted than observed total sugar intakes suggest that added sugars may have decreased across tertiles. Thus, the total sugar content of the diet may not appropriately reflect differences in diet quality.

Our observations that intakes of whole grains, fluid milk, and fruit increased across tertiles of daily dietary quality are consistent with studies by O’Neil et al. [6,7], who compared diet quality assessed using the Healthy Eating Index-2005 (HEI-2005) among American children and adults who consumed each of 12 different breakfast patterns, including no breakfast. They found that those consuming breakfasts with grains, cereals, lower-fat milk, and whole fruit/100% fruit juice had higher daily HEI-2005 scores compared with those who did not consume breakfast. However, they also identified other breakfast patterns for which daily HEI-2005 scores did not differ from those of breakfast non-consumers. Our data also demonstrate that breakfast consumption per se does not guarantee a high-quality diet: the mean NRF 9.3 scores in the lowest tertile of breakfast consumers (387 for children and 389 for adults) were lower than the mean score for all breakfast non-consumers of 439. 

Taken together, our data suggest that the consumption of breakfasts that include whole grains, fluid milk, and fruit contribute to higher daily diet quality. We found that the increases in mean serving size of these foods across tertiles were largely due to increases in the proportions of individuals consuming these foods, rather than to a larger serving size per individual consumer. However, this was not the case for other foods, where reductions in both the proportion of individuals consuming these foods and the mean intake per consumer were associated with higher diet quality.

### 4.5. Strengths and Limitations

The strengths of our study include the large, nationally representative sample, comprehensive assessment of breakfast intake, and the fact that we controlled for a number of important covariates. The limitations include that our analysis was based on only one day of dietary intake per respondent, which may not reflect habitual intakes. However, the fact that we observed a number of sociodemographic differences both between breakfast consumers and non-consumers and across tertiles of daily diet quality suggests that most individuals were classified appropriately. Moreover, misclassification would tend to attenuate true differences, as opposed to exaggerating them.

Although not a limitation per se, it should be noted that the purpose of using the NRF 9.3 score in the IBRI studies was to provide a harmonized approach across countries to identify features of higher-quality breakfasts, rather than to compare dietary quality between countries. While the NRF 9.3 has the advantage of being based on nutrients rather than food groups, the nutrient reference values used to determine the NRF 9.3 scores differ considerably between Europe and North America. In general, the daily values (DVs) in North America [24,46] are higher than the nutrient reference values (NRVs) used in Europe [47]. For example, DVs. range from being 12–15% higher than NRVs. for vitamin A, vitamin C, fiber, and magnesium and up to as much as 300% higher for vitamin D, which has a DV of 20 mcg compared with an NRV of 5 mcg. Because scores for each nutrient are expressed as percentages of the relevant DV or NRV, it follows that the same nutrient intake would lead to a higher score in Europe. As an extreme example, a vitamin D intake of 5 mcg per day would be scored as 100% in Europe but only 25% in North America. For that reason, comparisons of daily NRF 9.3 scores between IBRI countries using different reference values are not appropriate. Nevertheless, for the overall goal of the IBRI project [2], using the NRF 9.3 provided a harmonized approach to identify higher-quality breakfasts within each country. Thus, while the absolute NRF 9.3 score for a given diet would differ across countries, the score was used to rank individuals within each country according to diet quality, thus identifying higher- versus lower-quality diets in each country.

## 5. Conclusions

In this national sample of Canadians aged 6 years and above, 90% reported consuming breakfast. Breakfast consumers had higher intakes of energy and key nutrients, as well as higher diet quality as assessed using the NRF 9.3. Among breakfast consumers, breakfast provided a mean of 389 kcal and 21.6% of daily energy intake. Relative to its contribution to energy, breakfast provided higher amounts of fruit, whole grains, and fluid milk, as well as nutrients of concern in the Canadian diet such as fiber, calcium, and vitamin D. It also contributed higher amounts of total sugars. When breakfast consumers were classified into tertiles of daily dietary quality, breakfast energy intake did not differ across tertiles for either children or adults. However, breakfast intakes of key micronutrients, fiber, and total sugars increased across tertiles, and among adults, breakfast intakes of saturated fat and sodium decreased. Mean intakes of fruit, whole grains, and fluid milk also increased across tertiles, as did the proportion of individuals consuming these foods. Increasing the proportion of Canadians consuming these foods at breakfast could contribute to improved diet quality. 

## Figures and Tables

**Table 1:** Sociodemographic and lifestyle characteristics of Canadian breakfast consumers and non-consumers ^1^.

Characteristic	Children/Teens (6–17 Years)		Adults (≥18 Years)	
	Breakfast (*n* = 3934)	No Breakfast (*n* = 423)	Breakfast (*n* = 12,550)	No Breakfast (*n* = 1380)
Mean age (year)	11.4 ± 0.06	13.9 ± 0.2 *	49.7 ± 0.2	42.6 ± 0.9 *
Sex (% male)	51.5 ± 1.0	47.3 ± 3.8	49.0 ± 0.3	58.2 ± 2.5 *
Ethnicity (% Caucasian)	67.7 ± 1.4	65.8 ± 4.0	74.8 ± 1.0	74.1 ± 2.5
Supplement use in past 30 day (% yes)	36.6 ± 1.2	31.5 ± 4.4	48.1 ± 0.9	34.2 ± 2.4 *
Current smoker (% yes) ^2^	2.2 ± 0.4	7.6 ± 2.0 *	16.5 ± 0.7	36.9 ± 2.6 *
Education (% university grad) ^3^	44.2 ± 1.5	31.6 ± 3.9 *	39.4 ± 1.0	32.2 ± 2.7 *
Marital status (% married/cohabiting)	n/a	n/a	65.2 ± 0.9	54.0 ± 2.8 *
Food secure (% yes)	83.4 ± 1.0	80.8 ± 3.0	89.2 ± 0.5	82.8 ± 2.2 *
Body mass index (BMI; kg/m^2^)	n/a	n/a	27.2 ± 0.1	28.0 ± 0.5
BMI *z*-score for age and sex	0.43 ± 0.04	0.54 ± 0.1	n/a	n/a
Overweight/obese (% yes) ^4^	30.7 ± 1.3	39.0 ± 3.9 *	60.9 ± 1.1	66.5 ± 3.2
Urban residence (% yes)	81.2 ± 1.2	80.0 ± 3.8	83.0 ± 0.8	79.5 ± 2.3
Immigrant to Canada (% yes)	10.4 ± 0.8	15.9 ± 3.5	28.3 ± 1.0	20.1 ± 2.2 *

^1^ All the data are weighted to the Canadian population, but sample n’s are displayed. Values are displayed as means ± standard error (SE) or proportions ± SE; ^2^ For those aged 6–17 years, the data reflect prevalence among those aged ≥12 years, who were asked about smoking; ^3^ For those aged 6–17 years, the data reflect whether a member of the household is or is not a university graduate; ^4^ For those aged 6–17 years, based on BMI *z*-score for age and sex; for adults, based on BMI > 25, * *p* < 0.05 compared to breakfast consumers in the same age category.

**Table 2:** Daily energy and nutrient intakes of Canadian breakfast consumers and non-consumers ^1^.

Nutrient	All Ages		Children (6–12 Years)		Teens (13–17 Years)		Adults (18–54 Years)		Older Adults (55+ Years)	
	Breakfast (*n* = 16,484)	No Breakfast (*n* = 1803)	Breakfast (*n* = 2227)	No Breakfast (*n* = 104)	Breakfast (*n* = 1707)	No Breakfast (*n* = 319)	Breakfast (*n* = 6688)	No Breakfast (*n* = 963)	Breakfast (*n* = 5862)	No Breakfast (*n* = 417)
**Energy and Macronutrients**										
Energy (kcal)	1896 ± 13	1760 ± 49 ***	1854 ± 22	1661 ± 70	2102 ± 33	1791 ± 68 ***	1980 ± 21	1801 ± 66 **	1740 ± 18	1649 ± 76
CHO (g)	231 ± 2	204 ± 5 ***	256 ± 3	220 ± 11 ***	275 ± 4	232 ± 11	234 ± 3	207 ± 7 **	211 ± 2	188 ± 9 **
CHO (% energy)	48.8 ± 0.2	46.8 ± 0.5 **	55.0 ± 0.3	53.2 ± 1.2 ***	52.3 ± 0.4	50.9 ± 1.3	47.5 ± 0.3	46.6 ± 0.7	48.6 ± 0.2	45.3 ± 1.0 ***
Total sugars (g)	91.1 ± 0.8	85.2 ± 3.2	113 ± 1.9	99.1 ± 7.0	118 ± 2.6	97.1 ± 6.6	89.8 ± 1.3	87.7 ± 4.3	82.8 ± 1.2	73.5 ± 4.6 *
Fiber (g)	17.5 ± 0.2	12.1 ± 0.4 ***	16.0 ± 0.2	11.2 ± 0.7 ***	16.9 ± 0.3	12.7 ± 0.7 *	17.8 ± 0.2	12.1 ± 0.4 ***	17.6 ± 0.2	12.0 ± 0.8 ***
Fat (g)	69.9 ± 0.7	66.6 ± 2.5 *	64.0 ± 1.0	63.3 ± 3.5 ***	76.8 ± 1.7	64.4 ± 3.0	74.2 ± 1.0	68.8 ± 3.5 *	63.8 ± 1.0	61.5 ± 2.6
Fat (% energy)	31.9 ± 0.1	32.6 ± 0.4	30.1 ± 0.3	33.3 ± 1.2 ***	31.6 ± 0.3	32.4 ± 1.1	32.5 ± 0.2	32.6 ± 0.6	31.5 ± 0.2	32.6 ± 0.9
Saturates (g)	23.0 ± 0.2	22.1 ± 0.9	23.1 ± 0.4	22.5 ± 1.9	26.6 ± 0.6	21.1 ± 1.2	24.0 ± 0.4	22.8 ± 1.2	20.8 ± 0.3	20.6 ± 1.3
Monounsaturates (g)	26.0 ± 0.3	25.2 ± 1.1 **	22.7 ± 0.4	22.7 ± 1.4 **	28.1 ± 0.7	24.0 ± 1.2	27.8 ± 0.4	26.3 ± 1.5 **	23.6 ± 0.4	22.8 ± 1.5
Polyunsaturates (g)	14.7 ± 0.2	13.4 ± 0.6	12.4 ± 0.3	12.5 ± 0.8 **	15.1 ± 0.4	13.5 ± 0.6	15.6 ± 0.3	13.8 ± 0.8	13.7 ± 0.2	12.5 ± 1.0
Cholesterol (mg)	266 ± 4	238 ± 12	211 ± 5	170 ± 15	270 ± 8	199 ± 16 *	292 ± 7	252 ± 17	241 ± 4	217 ± 12
Protein (g)	79.2 ± 0.6	73.0 ± 2.3	69.0 ± 0.9	57.7 ± 3.2	83.4 ± 1.5	74.0 ± 6.9	85.2 ± 1.1	75.5 ± 3.2	71.7 ± 0.8	67.6 ± 3.0
Protein (% energy)	16.8 ± 0.1	16.7 ± 0.3	14.9 ± 0.1	13.5 ± 0.5	16.0 ± 0.2	16.6 ± 1.3	17.3 ± 0.1	16.8 ± 0.4	16.6 ± 0.1	17.1 ± 0.6
**Vitamins**										
Vitamin A (μg RAE)	645 ± 9	553 ± 37	600 ± 20	402 ± 34	664 ± 18	548 ± 97 ***	644 ± 13	567 ± 54	655 ± 15	530 ± 59
Thiamin (mg)	1.62 ± 0.01	1.32 ± 0.04 ***	1.64 ± 0.03	1.27 ± 0.08 *	1.83 ± 0.04	1.49 ± 0.10 **	1.68 ± 0.02	1.33 ± 0.05	1.49 ± 0.02	1.23 ± 0.07 **
Riboflavin (mg)	1.92 ± 0.01	1.70 ± 0.05 **	1.83 ± 0.03	1.39 ± 0.08	2.03 ± 0.04	1.51 ± 0.12 ***	2.01 ± 0.02	1.73 ± 0.07 *	1.80 ± 0.02	1.71 ± 0.08
Niacin (mg NE)	38.9 ± 0.3	36.6 ± 1.3	32.4 ± 0.4	26.9 ± 1.7	40.2 ± 0.8	37.6 ± 3.6	42.0 ± 0.6	38.3 ± 1.8	35.6 ± 0.4	32.5 ± 1.6
Vitamin B6 (mg)	1.67 ± 0.01	1.49 ± 0.06	1.40 ± 0.02	1.06 ± 0.09	1.67 ± 0.04	1.42 ± 0.16 **	1.78 ± 0.02	1.58 ± 0.08	1.57 ± 0.02	1.30 ± 0.09
Vitamin B12 (μg)	4.01 ± 0.06	3.99 ± 0.27	3.78 ± 0.19	2.68 ± 0.25	4.29 ± 0.14	2.90 ± 0.29 ***	4.14 ± 0.09	4.20 ± 0.39	3.80 ± 0.09	3.88 ± 0.36
Folate (μg DFE)	450 ± 4	382 ± 12 ***	446 ± 8	384 ± 29	492 ± 12	457 ± 44	478 ± 7	388 ± 14 ***	400 ± 5	343 ± 22 *
Folic acid (μg)	118 ± 2	106 ± 4 **	139 ± 3	123 ± 12	150 ± 5	148 ± 20	123 ± 3	104 ± 5) **	98 ± 2	96 ± 8
Vitamin C (mg)	103 ± 1.5	75 ± 5.8 ***	117 ± 3.0	90.4 ± 13.5 ***	119 ± 4.0	91.9 ± 12.4 *	104 ± 2.6	78.3 ± 8.2 ***	96.1 ± 2.1	60.4 ± 6.8 *
Vitamin D (μg)	4.88 ± 0.07	3.98 ± 0.29	5.27 ± 0.12	3.97 ± 0.60	5.71 ± 0.18	4.17 ± 0.56 ***	4.72 ± 0.11	3.65 ± 0.29	4.88 ± 0.11	4.82 ± 0.84
**Minerals and Electrolytes**										
Calcium (mg)	818 ± 8	691 ± 30	936 ± 15	749 ± 50	1005 ± 22	771 ± 55 **	828 ± 12	705 ± 42	738 ± 10	623 ± 33
Iron (mg)	12.7 ± 0.1	10.1 0.3 ***	12.6 ± 0.2	9.6 ± 0.6 ***	13.8 ± 0.3	11.4 ± 1.0 ***	13.0 ± 0.2	10.0 ± 0.3 ***	11.9 ± 0.1	10.0 ± 0.6
Magnesium (mg)	308 ± 2	242 ± 7 ***	254 ± 3	200 ± 11 **	297 ± 5	251 ± 24 **	322 ± 4	244 ± 8 ***	302 ± 3	2399 ± 11 ***
Potassium (mg)	2691 ± 19	2280 ± 63 **	2384 ± 30	1936 ± 123	2651 ± 45	2036 ± 120 ***	2763 ± 30	2281 ± 73 **	2667 ± 27	2385 ± 146
Sodium (mg)	2751 ± 24	2556 ± 84	2586 ± 38	2283 ± 136	2993 ± 58	2455 ± 111	2904 ± 39	2646 ± 111	2513 ± 30	2372 ± 158
Zinc (mg)	10.5 ± 0.1	9.4 ± 0.3	9.1 ± 0.1	7.6 ± 0.5	10.9 ± 0.2	9.2 ± 0.8 *	11.1 ± 0.2	9.6 ± 0.4	9.9 ± 0.2	9.3 ± 0.5
**Nutrient-Rich Foods (NRF) Index 9.3 Score ^2^**										
NRF 9.3	524 ± 2	439 ± 7 ***	516 ± 4	424 ± 16 *	499 ± 4	429 ± 16 ***	515 ± 3	433 ± 9 ***	546 ± 3	463 ± 17

^1.^ The data are shown as unadjusted mean ± SE and are weighted to the Canadian population. Sample sizes are unweighted. ^2.^ The Nutrient-Rich Foods Index 9.3 score reflects the sum of intakes (expressed as percentages of Canadian daily values (DVs) normalized to an intake of 2000 kcal (8.2 MJ) of 9 nutrients to encourage (protein, fiber, vitamins A, C, and D, calcium, iron, magnesium, and potassium) minus the sum of the percentages by which intakes of 3 nutrients to limit (total sugars, saturated fat, and sodium) exceed the DV. Percentage DVs. for nutrients to encourage are truncated at 100 for intakes ≥ DV, while percentage DVs. for nutrients to limit are not truncated at 100. Higher scores reflect higher diet quality; the maximum possible score is 900 (where intakes/2000 kcal for all 9 nutrients to encourage are >DV, and intakes of all 3 nutrients to limit are < DV). CHO: Carbohydrate; DFE: dietary folate equivalents; NE: niacin equivalents; RAE: retinol activity equivalents. *, **, ***: Intake differed between breakfast consumers and non-consumers in the same age group at *p* < 0.05, <0.01, and <0.001, respectively. The regression models included energy and covariates that differed significantly between breakfast consumers and non-consumers. However, models for energy, macronutrients expressed as a percentage of energy, and the NRF 9.3 score included only sociodemographic covariates, as energy adjustment was not applicable or was already considered in the calculation of % energy and the NRF 9.3 score.

**Table 3:** Energy and nutrient intakes at breakfast among Canadian breakfast consumers ^1^.

Nutrient	All 6+ Years (*n* = 16,484)	Children 6–12 Years (*n* = 2227)	Teens 13–17 Years (*n* = 1707)	Adults 18–54 Years (*n* = 6688)	Adults 55+ Years (*n* = 5862)	*p*
Energy (kcal)	389 ± 5	356 ± 8 ^L^	421 ± 10 ^H^	401 ± 8	374 ± 6	<0.001
CHO (g)	54.6 ± 0.6	53.8 ± 1.2 ^L^	59.7 ± 1.5 ^H^	54.2 ± 1.0	54.5 ± 0.8	<0.001
(% energy)	57.8 ± 0.3	60.8 ± 0.5 ^H^	58.6 ± 0.7	56.4 ± 0.5 ^L^	59.0 ± 0.4	<0.001
Total sugars (g)	23.0 ± 0.3	24.9 ± 0.7	27.0 ± 0.8 ^H^	22.9 ± 0.6	22.0 ± 0.4 ^L^	<0.001
(% energy)	26.4 ± 0.03	29.5 ± 0.1 ^H^	27.9 ± 0.1	26.1 ± 0.1	25.7 ± 0.1 ^L^	<0.001
Fiber (g)	4.4 ± 0.1	3.4 ± 0.1 ^L^	3.8 ± 0.1	4.3 ± 0.1	4.9 ± 0.1 ^H^	<0.001
Fat (g)	13.4 ± 0.3	10.6 ± 0.4 ^L^	14.1 ± 0.6	14.4 ± 0.5 ^H^	12.4 ± 0.4	<0.001
(% energy)	27.3 ± 0.3	25 ± 0.5 ^L^	26.9 ± 0.6	28.2 ± 0.4 ^H^	26.5 ± 0.4	<0.001
Saturates (g)	4.7 ± 0.1	4.3 ± 0.2	5.5 ± 0.2 ^H^	5.0 ± 0.2	4.2 ± 0.1 ^L^	<0.001
(% energy)	9.9 ± 0.1	10.3 ± 0.2	10.4 ± 0.3 ^H^	10.1 ± 0.2	9.4 ± 0.2 ^L^	0.002
Monounsaturates (g)	4.9 ± 0.1	3.6 ± 0.1 ^L^	5.0 ± 0.2	5.3 ± 0.2 ^H^	4.4 ± 0.2	<0.001
Polyunsaturates (g)	2.7 ± 0.1	1.8 ± 0.1 ^L^	2.6 ± 0.2	2.8 ± 0.1 ^H^	2.7 ± 0.1	<0.001
Cholesterol (mg)	77.4 ± 2.7	50.7 ± 3.1 ^L^	81.4 ± 6.6	90.0 ± 4.8 ^H^	64.4 ± 2.9	<0.001
Protein (g)	14.4 ± 0.2	12.5 ± 0.3 ^L^	15.4 ± 0.5 ^H^	15.4 ± 0.4 ^H^	13.2 ± 0.2	<0.001
(% energy)	14.9 ± 0.1	14.2 ± 0.2 ^L^	14.5 ± 0.2	15.3 ± 0.2 ^H^	14.4 ± 0.2	<0.001
Vitamin A (μg RAE)	149 ± 3	143 ± 4	180 ± 8 ^H^	156 ± 5	133 ± 4 ^L^	<0.001
Thiamin (mg)	0.44 ± 0.01	0.43 ± 0.01 ^L^	0.50 ± 0.02 ^H^	0.43 ± 0.01	0.44 ± 0.01	0.01
Riboflavin (mg)	0.57 ± 0.01	0.52 ± 0.02 ^L^	0.62 ± 0.02 ^H^	0.59 ± 0.01	0.53 ± 0.01	<0.001
Niacin (mg NE)	7.0 ± 0.1	5.6 ± 0.1 ^L^	7.1 ± 0.2	7.4 ± 0.2 ^H^	6.7 ± 0.1	<0.001
Vitamin B6 (mg)	0.31 ± 0.01	0.30 ± 0.01	0.34 ± 0.01	0.31 ± 0.01	0.31 ± 0.01	0.1
Vitamin B12 (μg)	0.89 ± 0.02	1.00 ± 0.03	1.15 ± 0.05 ^H^	0.94 ± 0.03	0.72 ± 0.02 ^L^	<0.001
Folate (μg DFE)	102.7 ± 1.4	95.1 ± 3.5 ^L^	110.1 ± 3.8 ^H^	107.2 ± 2.3	96.5 ± 2.0	<0.001
Folic acid (μg)	30.1 ± 0.5	33.8 ± 1.7	36.7 ± 1.9 ^H^	30.6 ± 0.9	27.4 ± 0.8 ^L^	<0.001
Vitamin C (mg)	21.2 ± 0.7	19.2 ± 1.2	22.0 ± 1.6	21.2 ± 1.2	21.6 ± 0.9	0.4
Vitamin D (μg)	1.67 ± 0.03	2.00 ± 0.07	2.22 ± 0.09 ^H^	1.65 ± 0.05	1.51 ± 0.04 ^L^	<0.001
Calcium (mg)	225 ± 3	270 ± 7	282 ± 9 ^H^	224 ± 5	206 ± 4 ^L^	<0.001
Iron (mg)	3.34 ± 0.04	3.47 ± 0.10	3.92 ± 0.16 ^H^	3.27 ± 0.06 ^L^	3.29 ± 0.07	0.001
Magnesium (mg)	77.3 ± 1.1	58.3 ± 1.3 ^L^	68.3 ± 2.1	78.5 ± 1.8	81.9 ± 1.7 ^H^	<0.001
Potassium (mg)	580 ± 7	477 ± 10 ^L^	558 ± 15	590 ± 12	594 ± 9 ^H^	<0.001
Sodium (mg)	494 ± 9	435 ± 12 ^L^	549 ± 22 ^H^	523 ± 15	453 ± 11	<0.001
Zinc (mg)	2.03 ± 0.03	1.78 ± 0.04 ^L^	2.16 ± 0.08 ^H^	2.10 ± 0.05	1.97 ± 0.04	<0.001

^1^ The data are displayed as unadjusted mean ± SE and are weighted to the Canadian population. The sample sizes are unweighted. When intake differed significantly across age groups, the Hsu multiple comparisons with the best method was used to identify the groups with the highest and lowest intakes (superscripts H and L in the table, respectively). CHO: carbohydrate; DFE: dietary folate equivalents; NE: niacin equivalents; RAE: retinol activity equivalents.

**Table 4:** Breakfast energy and nutrient intakes of Canadians by tertile of daily NRF Index 9.3 (NRF 9.3) score ^1,2^.

Breakfast Intake	Children/teens 6–17 Years (*n* = 3934)				Adults 18+ Years (*n* = 12,550)			
	Tertile of NRF 9.3 Score				Tertile of NRF 9.3 Score			
	1 (Low)	2 (Middle)	3 (High)	*p* ^3^	1 (Low)	2 (Middle)	3 (High)	*p* ^3^
Daily NRF 9.3 score ^2^	387 ± 3	512 ± 1	628 ± 2	n/a	389 ± 2	527 ± 1	665 ± 2	n/a
Energy (kcal)	386 ± 12	392 ± 11	368 ± 9	0.822	389 ± 10	400 ± 9	381 ± 10	0.351
Energy (% of day)	18.7 ± 0.0 ^L^	21.1 ± 0.1 ^H^	21.0 ± 0.0	<0.001	20.0 ± 0.0 ^L^	21.6 ± 0.0	24.0 ± 0.0 ^H^	0.004
Carbohydrate (g)	53.0 ± 1.7 ^L^	59.4 ± 1.9 ^H^	56.0 ± 1.4	0.020	49.8 ± 1.1 ^L^	55.8 ± 1.2	57.4 ± 1.2 ^H^	<0.001
Total sugars (g)	23.3 ± 0.9 ^L^	27.3 ± 1.1 ^H^	26.7 ± 0.7	0.003	20.0 ± 0.6 ^L^	23.5 ± 0.6	23.9 ± 0.6 ^H^	<0.001
Fiber (g)	2.9 ± 0.1 ^L^	3.6 ± 0.2	4.1 ± 0.2 ^H^	<0.001	3.2 ± 0.1 ^L^	4.4 ± 0.1	6.0 ± 0.2 ^H^	<0.001
Fat (g)	13.9 ± 0.7	11.8 ± 0.5	10.4 ± 0.4	0.108	15.4 ± 0.6 ^H^	14.1 ± 0.6	11.4 ± 0.5 ^L^	<0.001
Saturates (g)	5.4 ± 0.3	4.6 ± 0.2	4.0 ± 0.2	0.119	5.6 ± 0.2 ^H^	4.7 ± 0.2	3.7 ± 0.2 ^L^	<0.001
Monounsaturates (g)	4.8 ± 0.2	4.1 ± 0.2	3.6 ± 0.2	0.081	5.7 ± 0.3 ^H^	5.2 ± 0.3	4.1 ± 0.2 ^L^	<0.001
Polyunsaturates (g)	2.4 ± 0.2	2.1 ± 0.1	1.8 ± 0.1	0.491	2.8 ± 0.1	3.0 ± 0.1 ^H^	2.5 ± 0.1 ^L^	0.030
Cholesterol (mg)	72.1 ± 5.4	61.0 ± 4.9	56.1 ± 4.9	0.708	91.2 ± 5.4 ^H^	80.6 ± 5.4	67.9 ± 4.6 ^L^	0.039
Protein (g)	13.2 ± 0.5	13.4 ± 0.4	14.4 ± 0.4	0.131	14.2 ± 0.4 ^L^	14.4 ± 0.4	15.0 ± 0.4 ^H^	0.037
Vitamin A (μg RAE)	144 ± 7 ^L^	149 ± 6	181 ± 8 ^H^	0.019	129 ± 5 ^L^	146 ± 6	166 ± 6 ^H^	<0.001
Thiamin (mg)	0.42 ± 0.02 ^L^	0.44 ± 0.02	0.52 ± 0.02 ^H^	0.033	0.38 ± 0.01 ^L^	0.43 ± 0.02	0.49 ± 0.01 ^H^	<0.001
Riboflavin (mg)	0.51 ± 0.02 ^L^	0.55 ± 0.02	0.63 ± 0.02 ^H^	<0.001	0.54 ± 0.01 ^L^	0.57 ± 0.01	0.59 ± 0.01 ^H^	<0.001
Niacin (mg NE)	6.1 ± 0.2	6.2 ± 0.2	6.4 ± 0.2	0.265	7.0 ± 0.2	7.0 ± 0.2 ^L^	7.3 ± 0.2 ^H^	0.032
Vitamin B6 (mg)	0.25 ± 0.01 ^L^	0.34 ± 0.02	0.36 ± 0.01 ^H^	<0.001	0.25 ± 0.01 ^L^	0.30 ± 0.01	0.38 ± 0.01 ^H^	<0.001
Vitamin B12 (μg)	0.90 ± 0.04 ^L^	1.05 ± 0.05	1.22 ± 0.05 ^H^	<0.001	0.78 ± 0.04 ^L^	0.84 ± 0.03	0.95 ± 0.03 ^H^	<0.001
Folate, DFE (μg)	101.6 ± 4.6	106.4 ± 4.9	95.4 ± 3.8	0.436	101.6 ± 2.9	104.6 ± 2.7	102.8 ± 2.7	0.195
Vitamin C (mg)	16.2 ± 1.6	24.0 ± 2.0	20.7 ± 1.5	0.051	12.6 ± 1.0 ^L^	23.6 ± 1.6	27.8 ± 1.4 ^H^	<0.001
Vitamin D (μg)	1.7 ± 0.1 ^L^	2.0 ± 0.1	2.5 ± 0.1 ^H^	<0.001	1.3 ± 0.1 ^L^	1.7 ± 0.1	1.8 ± 0.1 ^H^	<0.001
Calcium (mg)	221 ± 8 ^L^	274 ± 11	328 ± 10 ^H^	<0.001	172 ± 6 ^L^	218 ± 6	262 ± 8 ^H^	<0.001
Iron (mg)	3.1 ± 0.1 ^L^	3.8 ± 0.2	4.1 ± 0.1 ^H^	<0.001	2.9 ± 0.1 ^L^	3.2 ± 0.1	3.8 ± 0.1 ^H^	<0.001
Magnesium (mg)	51.9 ± 1.7 ^L^	62.6 ± 2.2	72.4 ± 2.4 ^H^	<0.001	63.8 ± 2.0 ^L^	79.6 ± 2.5	96.1 ± 2.3 ^H^	<0.001
Potassium (mg)	426 ± 14 ^L^	512 ± 16	590 ± 17 ^H^	<0.001	488 ± 12 ^L^	592 ± 14	695 ± 15 ^H^	<0.001
Sodium (mg)	540 ± 26	479 ± 16	424 ± 13	0.075	572 ± 19 ^H^	492 ± 14	423 ± 17 ^L^	<0.001
Zinc (mg)	1.7 ± 0.1 ^L^	1.9 ± 0.1	2.2 ± 0.1 ^H^	0.003	1.8 ± 0.1 ^L^	2.0 ± 0.1	2.3 ± 0.1 ^H^	<0.001

^1^ The data are shown as unadjusted mean ± SE and are weighted to the Canadian population. Sample sizes are unweighted; ^2^ The Nutrient-Rich Foods Index 9.3 score reflects the sum of daily intakes expressed as percentages of Canadian daily values (DVs) normalized to an intake of 2000 kcal (8.2 MJ) of 9 nutrients to encourage (protein, fiber, vitamins A, C, and D, calcium, iron, magnesium, and potassium) minus the sum of the percentages by which intakes of 3 nutrients to limit (total sugars, saturated fat, and sodium) exceed the DV. Percentage DVs. for nutrients to encourage are truncated at 100 for intakes >DV, while percentage DVs. for nutrients to limit are not truncated at 100. The maximum possible score is 900 (where intakes/2000 kcal for all 9 nutrients to encourage are >DV, and intakes of all 3 nutrients to limit are <DV); ^3^ The regression models included sociodemographic covariates that differed significantly across the NRF 9.3 tertiles. For children, these were age, parental education, food security status, and urban residence. For adults, age, sex, ethnicity, supplement use, education, food security status, BMI, and immigration status were included. When intake differed significantly across tertiles, the Hsu multiple comparisons with the best method was used to identify the groups with the highest and lowest intakes (superscript H and L in the table, respectively). DFE: dietary folate equivalents; NE: niacin equivalents; and RAE: retinol activity equivalents.

**Table 5:** Mean food group intake at breakfast and proportions consuming food groups at breakfast by the daily Nutrient-Rich Foods Index 9.3 tertile among Canadian children/teens and adults ^1^.

**Children/Teens 6–17 Years (*n* = 3934)**								
**Food Group ^2^**	**Mean Food Group Intake (Servings or kcal ^3^)**			***p*^4^**	**Proportion Consuming Food Group (%)**			***p***
	**Tertile 1**	**Tertile 2**	**Tertile 3**		**Tertile 1**	**Tertile 2**	**Tertile 3**	
*Vegetables and Fruit*	0.41 ± 0.0 ^L^	0.61 ± 0.04	0.61 ± 0.04 ^H^	0.006	28.4 ± 2.1 ^L^	36.8 ± 2.1	39.3 ± 2.1 ^H^	<0.001
Whole Fruit	0.13 ± 0.02 ^L^	0.21 ± 0.02	0.29 ± 0.03 ^H^	<0.001	13.2 ± 1.5 ^L^	18.1 ± 1.6	25.6 ± 2.0 ^H^	<0.001
Fruit Juice	0.18 ± 0.02 ^L^	0.33 ± 0.03 ^H^	0.27 ± 0.03	<0.001	10.2 ± 1.3 ^L^	17.5 ± 1.5 ^H^	14.6 ± 1.5	0.002
*Grain Products*	1.50 ± 0.07	1.56 ± 0.06	1.48 ± 0.05	0.430	83.0 ± 1.7 ^L^	89.0 ± 1.3	89.6 ± 1.3 ^H^	0.001
Whole Grains	0.23 ± 0.03 ^L^	0.45 ± 0.04	0.66 ± 0.05 ^H^	<0.001	16.6 ± 1.6 ^L^	29.3 ± 1.9	43.2 ± 2.3 ^H^	<0.001
Non-Whole Grains	1.26 ± 0.07 ^H^	1.11 ± 0.06	0.82 ± 0.05 ^L^	0.002	68.3 ± 2.0 ^H^	62.7 ± 2.0	50.8 ± 2.2 ^L^	<0.001
*Milk and Alternatives*	0.46 ± 0.02 ^L^	0.57 ± 0.03	0.76 ± 0.03 ^H^	<0.001	58.3 ± 2.2 ^L^	64.5 ± 2.1	78.4 ± 1.9 ^H^	<0.001
Fluid Milk	0.35 ± 0.0 ^L^	0.49 ± 0.02	0.70 ± 0.03 ^H^	<0.001	47.7 ± 2.1 ^L^	59.5 ± 2.1	75.3 ± 1.9 ^H^	<0.001
Other Milk Products	0.11 ± 0.01 ^H^	0.08 ± 0.01	0.06 ± 0.01 ^L^	0.025	16.2 ± 1.6 ^H^	10.1 ± 1.1	9.9 ± 1.3 ^L^	<0.001
*Meat and Alternatives*	0.20 ± 0.02	0.18 ± 0.02	0.17 ± 0.02	0.508	26.8 ± 2.0	24.9 ± 1.8	23.9 ± 2.0	0.580
*Other Foods*	61.8 ± 6.0 ^H^	48.0 ± 3.9	25.2 ± 2.0 ^L^	0.019	67.7 ± 2.1 ^H^	60.6 ± 2.1	49.5 ± 2.1 ^L^	<0.001
**Adults 18+ years (*n* = 12,550)**								
**Food Group ^2^**	**Mean Food Group Intake (Servings or kcal ^3^)**			***p*** **^4^**	**Proportion Consuming Food Group (%)**			***p***
	**Tertile 1**	**Tertile 2**	**Tertile 3**		**Tertile 1**	**Tertile 2**	**Tertile 3**	
*Vegetables and Fruit*	0.42 ± 0.03 ^L^	0.72 ± 0.04	0.96 ± 0.04 ^H^	<0.001	29.9 ± 1.4 ^L^	45.3 ± 1.6	56.2 ± 1.4 ^H^	<0.001
Whole Fruit	0.18 ± 0.02 ^L^	0.37 ± 0.03	0.54 ± 0.03 ^H^	<0.001	15.8 ± 1.0 ^L^	30.0 ± 1.5	41.5 ± 1.4 ^H^	<0.001
Fruit Juice	0.16 ± 0.02 ^L^	0.26 ± 0.03	0.28 ± 0.03 ^H^	<0.001	8.4 ± 1.0 ^L^	13.8 ± 1.2	13.9 ± 1.0 ^H^	<0.001
*Grain Products*	1.49 ± 0.04	1.53 ± 0.04	1.47 ± 0.04	0.296	78.1 ± 1.2 ^L^	82.8 ± 1.2	83.4 ± 1.1 ^H^	0.003
Whole Grains	0.33 ± 0.02 ^L^	0.52 ± 0.03	0.68 ± 0.03 ^H^	<0.001	20.0 ± 1.3 ^L^	32.2 ± 1.3	44.0 ± 1.5 ^H^	<0.001
Non-Whole Grains	1.17 ± 0.04 ^H^	1.02 ± 0.04	0.79 ± 0.04 ^L^	<0.001	60.6 ± 1.5 ^H^	55.3 ± 1.5	45.8 ± 1.4 ^L^	<0.001
*Milk and Alternatives*	0.29 ± 0.01 ^L^	0.42 ± 0.02	0.50 ± 0.02 ^H^	<0.001	50.1 ± 1.6 ^L^	62.1 ± 1.6	67.9 ± 1.4 ^H^	<0.001
Fluid Milk	0.18 ± 0.01 ^L^	0.30 ± 0.02	0.38 ± 0.02 ^H^	<0.001	40.5 ± 1.6 ^L^	53.5 ± 1.6	58.8 ± 1.4 ^H^	<0.001
Other Milk Products	0.11 ± 0.01	0.11 ± 0.01	0.12 ± 0.01	0.452	17.0 ± 1.1	17.3 ± 1.3	17.5 ± 1.1	0.958
*Meat and Alternatives*	0.31 ± 0.02	0.32 ± 0.02	0.28 ± 0.01	0.835	36.6 ± 1.7	36.8 ± 1.5	36.9 ± 1.5	0.989
*Other Foods*	74.1 ± 3.9 ^H^	54.4 ± 3.9	37.8 ± 2.8 ^L^	<0.001	74.2 ± 1.4 ^H^	67.1 ± 1.5	65.2 ± 1.4 ^L^	<0.001

^1^ The data are shown as unadjusted means ± SE or proportions ± SE and are weighted to the Canadian population; ^2^ Major food groups are italicized; food subgroups appear below major food groups and not italicized; ^3^ Mean numbers of servings averaged across all individuals in a tertile are shown for all food groups except other foods, which are displayed in mean kcal; ^4^ For mean intakes, the regression models included sociodemographic covariates that differed significantly across the NRF 9.3 tertiles. For children, these were age, parental education, food security status, and urban residence. For adults, age, sex, ethnicity, supplement use, education, food security status, BMI, and immigration status were included. When values differed significantly across tertiles, the Hsu multiple comparisons with the best method was used to identify the groups with the highest and lowest intakes (superscripts H and L in the table, respectively).

## Supplementary Materials

The following are available online at http://www.mdpi.com/2072-6643/10/8/985/s1. Figure S1: Contribution of nutrient intakes at breakfast to total daily intakes of Canadian children (6–12 years) and adolescent (13–17 years) breakfast consumers; Figure S2: Contribution of nutrient intakes at breakfast to total daily intakes of Canadian younger (18–54 years) and older (55+ years) adult breakfast consumers; Table S1: Proportions consuming food groups and mean percentages of energy and nutrient intakes at breakfast provided by food groups; and Table S2: Sociodemographic and lifestyle characteristics of children/teens and adults by daily NRF 9.3 tertile.
