# Correction: Breakfast in Canada: Prevalence of Consumption, Contribution to Nutrient and Food Group Intakes, and Variability across Tertiles of Daily Diet Quality. A Study from the International Breakfast Research Initiative. *Nutrients* 2018, *10*, 985

**DOI:** 10.3390/nu12092675

**Published:** 2020-09-02

**Authors:** Susan I. Barr, Hassan Vatanparast, Jessica Smith

**Affiliations:** 1 Department of Food, Nutrition and Health, University of British Columbia, FNH 244, 2205 East Mall, Vancouver, BC V6T 1Z4, Canada; 2 College of Pharmacy and Nutrition, School of Public Health, University of Saskatchewan, Saskatoon, SK S7N 4Z2, Canada; vatan.h@usask.ca; 3 Bell Institute of Health and Nutrition, General Mills, Minneapolis, MN 55427-3870, USA; Jessica.Smith@genmills.com

The authors wish to make a correction to the published version of their paper [1]. The words “lower intakes of” should be deleted from the final sentence of text Section 3.2: Daily Intakes of Breakfast Consumers and Non-Consumers. Thus, the sentence should be “Teens aged 13–17 years had the greatest number of differences between consumers and non-consumers: in addition to a higher NRF 9.3 score, breakfast consumers had higher intakes of fiber, vitamin A, thiamin, riboflavin, vitamin B6, vitamin B12, vitamin C, vitamin D, calcium, iron, magnesium, potassium, and zinc, as well as cholesterol”. We apologize for this error. The change does not affect the scientific results. The published version will be updated on the article webpage, with a reference to this correction notice.

## References

[1] Barr S.I., Vatanparast H., Smith J. (2018). Breakfast in Canada: Prevalence of Consumption, Contribution to Nutrient and Food Group Intakes, and Variability across Tertiles of Daily Diet Quality. A Study from the International Breakfast Research Initiative. Nutrients.

