# Supplementary material for: Breakfast in Canada: Prevalence of Consumption, Contribution to Nutrient and Food Group Intakes, and Variability across Tertiles of Daily Diet Quality. A Study from the International Breakfast Research Initiative
Source: Nutrients. 2018 Jul 27;10(8):985. doi: 10.3390/nu10080985 (PMC6116091; doi:10.3390/nu10080985)
Supplement: Supplementary file 1 [file nutrients-10-00985-s001.pdf]

Supplementary Materials

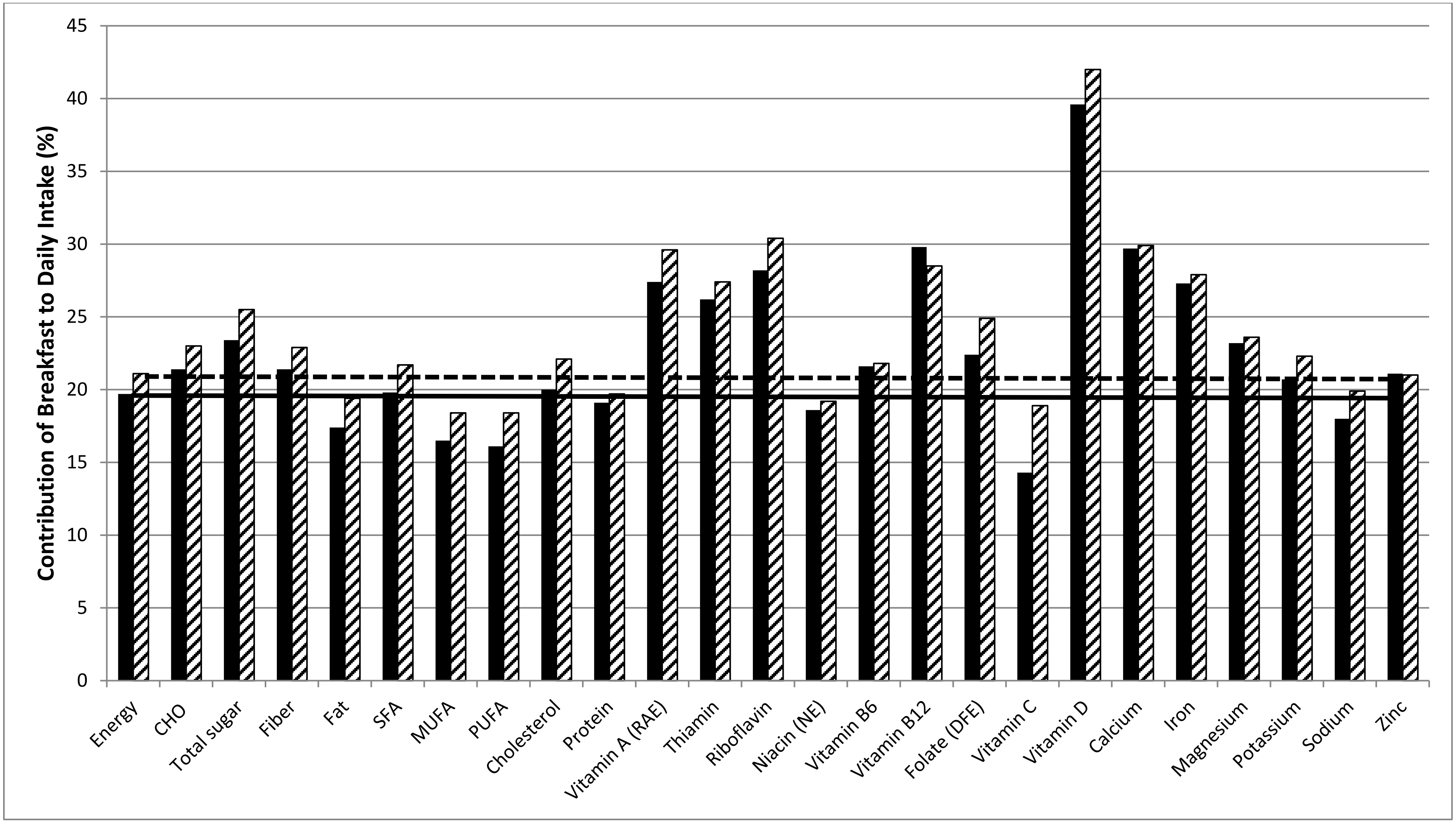

Figure S1. Contribution of nutrient intakes at breakfast to total daily intakes of Canadian children (6–12 y, ■) and adolescent (13–17 y, ▨) breakfast consumers. The horizontal lines represent the percentage of daily energy intake consumed at breakfast.

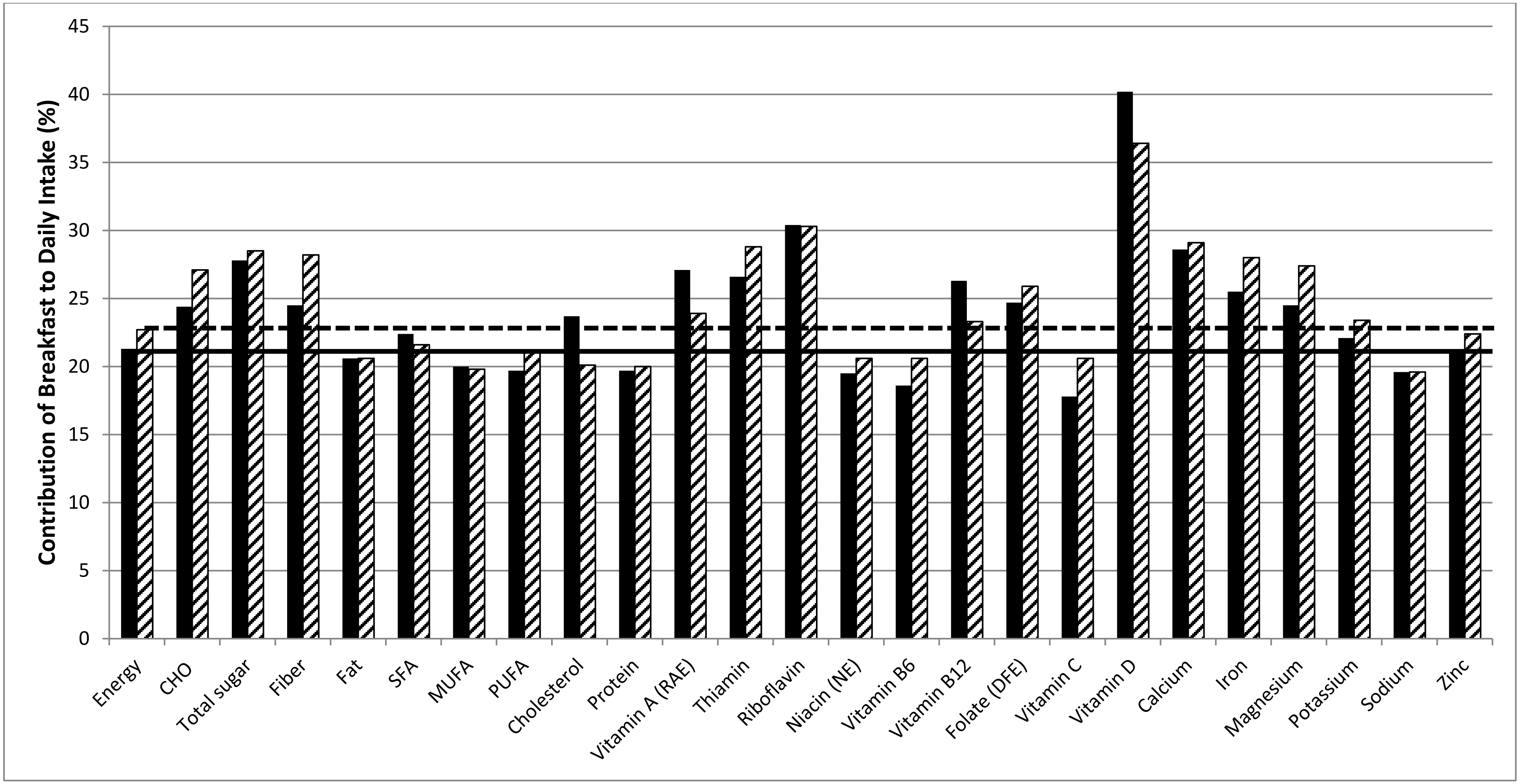

Figure S2. Contribution of nutrient intakes at breakfast to total daily intakes of Canadian younger (18–54 y, ■) and older (55+ y, ▨) adult breakfast consumers . The horizontal lines represent the proportion of daily energy intake consumed at breakfast.

Table S1. Proportions consuming food groups at breakfast and mean percentages of breakfast energy and nutrient intakes from food groups.

Food Group	Percent Consumers	Percentage of Breakfast Energy or Nutrient Intake											
		Energy	Total sugars	Sat fat	Fiber	Folate	Vit A	Vit D	Ca	Fe	Na	K	Mg
**Children and Teens (6–17 y, n = 3934)**													
Fruit juice	14.1 ± 0.8[1]	3.9	7.2	0.7	3.5	3.9	2.6	0.4	3.1	2.0	0.7	7.0	4.7
Whole fruit	19.0 ± 1.0	4.9	7.9	2.0	9.8	5.0	4.2	0.0	2.5	3.9	1.1	7.6	5.4
Whole grains	29.7 ± 1.1	10.0	6.1	5.8	17.2	13.0	0.2	2.0	5.6	17.2	9.4	5.8	10.7
Non-whole grains	60.6 ± 1.2	24.6	16.9	10.5	39.2	37.2	4.5	10.4	14.3	40.0	30.0	13.4	21.8
Milk and Alternatives	67.1 ± 1.2	21.2	29.4	38.5	1.8	11.8	50.1	50.8	45.5	4.2	21.3	35.2	25.4
Meat and Alternatives	25.2 ± 1.1	6.8	2.8	10.0	2.8	7.2	6.4	6.4	3.9	6.9	5.2	6.2	6.2
Other Foods	59.2 ± 1.2	9.8	11.2	16.3	3.2	1.5	13.6	10.1	5.5	4.5	10.5	4.7	5.4
Total	n/a	80.9	81.6	83.9	77.6	79.5	81.6	80.1	80.3	78.7	78.3	79.9	79.7
**Adults (18+ y, n = 12,550)**													
Fruit juice	12.0 ± 0.6	2.8	5.5	0.5	2.2	3.5	2.2	0.8	2.5	1.5	0.4	4.9	3.1
Whole fruit	29.1 ± 0.8	7.9	13.5	3.3	14.5	8.5	7.4	0.0	3.9	6.0	1.5	11.3	7.9
Whole grains	32.1 ± 0.8	9.4	4.3	6.5	15.2	9.9	0.6	0.7	5.0	15.1	6.8	5.4	10.1
Non-whole grains	53.9 ± 0.9	21.2	15.0	9.6	34.8	30.6	2.3	10.6	15.6	33.4	25.8	11.1	19.8
Milk and Alternatives	60.0 ± 1.0	13.0	18.0	24.9	1.1	7.0	35.2	36.3	32.1	2.6	15.9	16.7	11.9
Meat and Alternatives	36.7 ± 1.0	9.6	3.9	14.4	4.8	9.3	8.5	9.0	5.9	9.3	7.1	8.1	8.6
Other Foods	68.8 ± 0.9	14.1	17.5	21.1	2.6	2.0	21.0	16.8	7.7	4.3	14.2	4.9	5.2
Total	n/a	77.9	77.7	80.3	75.1	70.7	77.2	74.2	72.6	72.3	71.9	62.5	66.6

[1.] Data for percent consumers are shown as mean percentages ± Standard Error (SE); data for energy and nutrients are shown as mean percentages of breakfast intake. SEs for the percentage of nutrient intake provided by each of the food groups at breakfast were all <0.1% and so are not displayed.

Table S2. Sociodemographic and lifestyle characteristics of children/teen and adult breakfast consumers by daily Nutrient Rich Food index 9.3 tertile[1,2].

Characteristic	Children/teens (6–17y; n = 3934)				Adults (≥18y; n = 12,550)			
	1 (low)	2 (middle)	3 (high)	P	1 (low)	2 (middle)	3 (high)	P
	Mean or % ± SE	Mean or % ± SE	Mean or % ± SE		Mean or % ± SE	Mean or % ± SE	Mean or % ± SE	
Mean age (y)	11.7 ± 0.1	11.5 ± 0.1	10.9 ± 0.1	0.001	47.8 ± 0.4	48.5 ± 0.5	52.7 ± 0.4	<0.001
Sex (% Female)	47.5 ± 2.0	52.0 ± 2.0	46.0 ± 2.3	0.167	42.6 ± 1.4	50.3 ± 1.4	60.0 ± 1.2	<0.001
Sex (% male)	52.5 ± 2.0	48.0 ± 2.0	54.0 ± 2.3	0.167	57.4 ± 1.4	49.7 ± 1.4	40.0 ± 1.2	<0.001
Ethnicity (% Caucasian)	69.9 ± 2.3	64.0 ± 2.6	69.4 ± 2.2	0.128	79.0 ± 1.4	72.4 ± 1.7	73.2 ± 1.5	0.001
Supplement use (% yes)	34.5 ± 2.0	35.7 ± 2.1	39.8 ± 2.3	0.188	38.9 ± 1.5	47.9 ± 1.5	57.4 ± 1.5	<0.001
Smoker (% yes)	3.9 ± 0.8	1.3 ± 0.6	1.1 ± 0.6	0.013	23.5 ± 1.4	15.9 ± 1.2	10.0 ± 0.8	<0.001
Education (% university grad)	35.5 ± 2.2	47.2 ± 2.4	49.8 ± 2.3	<0.001	32.3 ± 1.5	41.8 ± 1.7	43.9 ± 1.5	<0.001
Marital status (% married/cohabiting)	--	--	--	n/a	62.1 ± 1.5	64.5 ± 1.5	66.9 ± 1.4	0.076
Food secure (% yes)	78.3 ± 2.0	83.2 ± 1.7	88.8 ± 1.3	<0.001	85.7 ± 1.1	90.2 ± 0.9	91.9 ± 0.8	<0.001
Body mass index (BMI; kg/m^2)	--	--	--	n/a	27.8 ± 0.2	27.0 ± 0.2	26.9 ± 0.2	0.002
BMI z-score	0.49 ± 0.07	0.44 ± 0.07	0.39 ± 0.07	0.598	--	--	--	n/a
Overweight/obese (% yes)	30.8 ± 2.1	31.0 ± 2.2	30.2 ± 2.4	0.968	63.9 ± 1.8	59.9 ± 1.9	59.0 ± 1.7	0.113
Urban residence (% yes)	77.8 ± 2.0	80.6 ± 1.9	85.1 ± 1.6	0.010	80.9 ± 1.2	84.1 ± 1.3	83.9 ± 1.2	0.087
Immigrant to Canada (% yes)	11.4 ± 1.5	11.4 ± 1.5	8.5 ± 1.3	0.293	23.2 ± 1.4	28.1 ± 1.5	33.3 ± 1.5	<0.001
Daily energy intake (kcal)	2153 ± 38	1967 ± 36	1786 ± 29	<.0001	2084 ± 28	1943 ± 27	1662 ± 21	<0.001
Breakfast energy intake (kcal)	386 ± 12	392 ± 11	368 ± 9	0.288	390 ± 10	400 ± 9	381 ± 10	0.280

[1] The Nutrient Rich Food index 9.3 score reflects the sum of daily intakes (expressed as percentages of Canadian Daily Values (DVs) normalized to an intake of 2000 kcal) of 9 nutrients to encourage (protein, fiber, vitamins A, C, and D, calcium, iron, magnesium, and potassium) minus the sum of the percentages by which intakes of the 3 nutrients to limit (total sugars, saturated fat, and sodium) exceed the DV. Higher scores reflect higher diet quality. [2] Data are shown as unadjusted means ± SE or proportions ± SE and are weighted to the Canadian population. Samples sizes are not weighted.
